# Suppression of a cold-sensitive mutation in ribosomal protein S5 reveals a role for RimJ in ribosome biogenesis

**DOI:** 10.1111/j.1365-2958.2008.06252.x

**Published:** 2008-06

**Authors:** Biswajoy Roy-Chaudhuri, Narayanaswamy Kirthi, Teresa Kelley, Gloria M Culver

**Affiliations:** 1 Department of Biology, University of RochesterRochester, NY 14627, USA; 2 Department of Biochemistry, Biophysics and Molecular Biology, Iowa State UniversityAmes, IA 50011, USA

## Abstract

A specific mutation of *Escherichia coli* ribosomal protein S5, in which glycine is changed to aspartate at position 28 [S5(G28D)], results in cold sensitivity and defects in ribosome biogenesis and translational fidelity. In an attempt to understand the roles of S5 in these essential cellular functions, we selected extragenic suppressors and identified *rimJ* as a high-copy suppressor of the cold-sensitive phenotype associated with the S5(G28D) mutation. Our studies indicate that RimJ overexpression suppresses the growth defects, anomalous ribosome profiles and mRNA misreading exhibited by the S5(G28D) mutant strain. Although previously characterized as the *N*-acetyltransferase of S5, our data indicate that RimJ, when devoid of acetyltransferase activity, can suppress S5(G28D) defects thus indicating that the suppression activity of RimJ is not dependent on its acetyltransferase activity. Additionally, RimJ appears to associate with pre-30S subunits indicating that it acts on the ribonucleoprotein particle. These findings suggest that RimJ has evolved dual functionality; it functions in r-protein acetylation and as a ribosome assembly factor in *E. coli*.

## Introduction

Ribosome biogenesis in *Escherichia coli* (*E. coli*) is a highly complex and co-ordinated process that remains largely uncharacterized despite its importance to cell physiology. The ribosomal RNAs (rRNAs) are synthesized as a primary transcript, which must be processed, folded and bound by ribosomal proteins (r-proteins) to form functional ribosomes (Wimberly *et al*., 2000). In *E. coli*, the two functionally distinct and asymmetric subunits that form the 70S, or the translationally active, ribosome are the small (30S) and large (50S) subunits. The small subunit contains a 16S rRNA and 21 r-proteins (S1-S21), while the large subunit contains two rRNAs, 5S and 23S, as well as 34 r-proteins. Thus, the process of ribosome biogenesis minimally involves the appropriate folding and interaction of over 50 r-proteins and three rRNAs (composed of more than 4000 nucleotides).

Although some bacterial ribosomal subunits self-assemble *in vitro* (Held *et al*., 1973), the non-physiological requirements (high temperature, high ionic conditions, long incubation and slow kinetics) (Traub and Nomura, 1968) suggest that additional factors work *in vivo* to facilitate ribosome biogenesis (Nierhaus *et al*., 1973; Culver, 2003). At least 170 non-ribosomal factors are thought to be involved in ribosome biogenesis in yeast (Fromont-Racine *et al*., 2003; Dez and Tollervey, 2004). In contrast, our knowledge of *E. coli* extra-ribosomal assembly factors is more limited (Hage and Tollervey, 2004). Currently, a few more than a dozen proteins have been implicated in aiding ribosome assembly in *E. coli.* These include chaperones (DnaK, RbfA, RimM; Bylund *et al*., 1998; Maki *et al*., 2002; Xia *et al*., 2003), GTPases (Era, Der, Cgt_E_; Inoue *et al*., 2003; Sharma *et al*., 2005; Hwang and Inouye, 2006; Jiang *et al*., 2006), RNA-remodeling proteins such as RNA helicases (DeaD, SrmB, DbpA; Pugh *et al*., 1999; Charollais *et al*., 2003; 2004), and RNA-modifying enzymes such as methyltransferases (KsgA, RrmJ; Lu and Inouye, 1998; Caldas *et al*., 2000) and pseudouridine synthases (RluD; Gutgsell *et al*., 2005). Thus while some of the enzymes responsible for rRNA modifications have been associated with ribosome biogenesis (Caldas *et al*., 2000; Gutgsell *et al*., 2005), the role of enzymes modifying r-proteins in ribosome biogenesis has not been established.

In a previous study, we identified a single mutation in S5 that confers spectinomycin resistance and cold sensitivity to *E. coli* (Kirthi *et al*., 2006). In the context of the large macromolecular complex (the 30S subunit), the mutation was shown to have profound effects on ribosome function, specifically tRNA binding and translational fidelity. Interestingly, this mutation is distinct from previously identified ribosome ambiguity (ram) mutations (Ito and Wittmann, 1973; Piepersberg *et al*., 1975a) and thus offers new insight into the role of S5 in maintenance of translational fidelity. Additionally, this S5 mutant strain exhibits defects in ribosome biogenesis, which is associated with the cold-sensitive phenotype and this type of defect has not been reported for the other S5 ram strains (Piepersberg *et al*., 1975a). To appreciate the basis of the functional defects of the S5(G28D) cold sensitivity in ribosome biogenesis, we selected for extragenic suppressors of the cold-sensitive phenotype using an *E. coli* genomic library.

Here, we describe the identification and characterization of a role of RimJ (Cumberlidge and Isono, 1979; Janda *et al*., 1985) in ribosome biogenesis. Our data suggest that RimJ, when overexpressed, suppresses the cold-sensitive, translational fidelity and ribosome biogenesis defects associated with S5(G28D). We have also shown that the S5(G28D) mutation in combination with *rimJ* deletion results in synthetic enhancement of the defective growth phenotype of the S5(G28D) strain. Additionally, we have shown that the suppressor function is independent of its acetyltransferase activity. We also demonstrate that RimJ associates with pre-30S subunits and can immunoprecipitate some 30S subunit components, including 16S rRNA, indicating that it exerts its function at the level of the RNP. Thus, an additional *in vivo* role has been identified for RimJ and leads to the proposal that RimJ acts as both a ribosome assembly factor and an r-protein modification enzyme.

## Results

### *rimJ* acts as a high-copy extragenic suppressor of S5(G28D) cold sensitivity

In an earlier study, the specific mutation of glycine to aspartate at position 28 in r-protein S5 was shown to result in spectinomycin resistance and cold sensitivity, whereby, at a reduced temperature (20°C), growth was undetectable for the mutant strain (Kirthi *et al*., 2006). To further explore the functional defects of the S5(G28D) strain, we isolated extragenic suppressors which would rescue the S5(G28D) cold-sensitive phenotype. A high-copy pUC-based *E. coli* genomic library was transformed into the S5(G28D) strain and colonies that could grow at the non-permissive temperature (20°C) were identified. A total of 24 independent isolates were subsequently re-screened for growth at the non-permissive temperature (data not shown). A third round of screening, where plasmid DNA isolated from each of the cold-tolerant colonies was transformed again into S5(G28D) cells, resulted in the identification of five independent suppressors.

To identify the gene(s) responsible for the suppression of the cold sensitivity of the S5(G28D) strain, plasmid DNA from each of the five isolates was sequenced from both ends of the insert. Sequence analysis revealed that all of the isolates contained overlapping DNA fragments (data not shown). Although DNA sequences on either side of the overlapping elements were unique, all suppressor DNA fragments contained the entire *rimJ* gene. The *rimJ* gene is of particular interest as its product, RimJ, has been shown to acetylate the N-terminal alanine of S5 (Yoshikawa *et al*., 1987) and hence, we speculated that RimJ was the likely candidate as the suppressor. To confirm that extra copies of *rimJ* and thus possible overexpression of RimJ is responsible for suppression of the cold-sensitive S5 mutation, the *rimJ* gene was cloned into a vector (pBAD-DEST49; p*-rimJ*) that allows regulated expression of the gene. Induced expression of *rimJ* from p*-rimJ* alleviates the cold-sensitive phenotype of S5(G28D) thereby confirming that *rimJ* can act as an extragenic suppressor.

### Overexpression of RimJ alleviates the growth defects associated with S5(G28D)

**Fig. 1:**
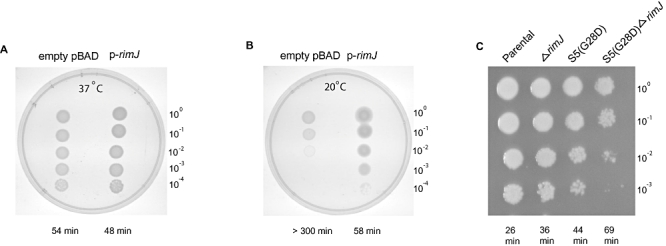
Analysis of growth of S5(G28D) strain in the presence and absence of RimJ. A and B. Growth of S5(G28D) cells in the absence or presence of RimJ induction as shown by the 10-fold serial dilutions of S5(G28D) with pBAD vector (empty pBAD) or with the same vector containing the *rimJ* gene for overexpression (p-*rimJ*) at the permissive, 37°C (A) and non-permissive temperature, 20°C (B). Plates contain arabinose to allow continued expression of *rimJ*. C. Wild-type parental (BW25113), Δ*rimJ* (JW1053), S5(G28D) and the double mutant [S5(G28D) Δ*rimJ*] are grown at the permissive, 37°C on LB plates. Corresponding doubling times in minutes for growth in liquid LB media (with appropriate antibiotics or arabinose) are also indicated for each strain at the bottom.

Overexpression of RimJ decreases the doubling time of S5(G28D) cells at all temperatures, although, the effect is more pronounced at the non-permissive temperature. At low temperature, the S5(G28D) strain has a doubling time at least five times longer than its parental strain and is virtually immeasurable (Kirthi *et al*., 2006; Fig. 1). Overproduction of RimJ in the S5 mutant strain reduces the doubling time to 58 min at 20°C (Fig. 1B), which is a comparable doubling time to the parental strain at low temperature (Kirthi *et al*., 2006). While at 37°C, the S5(G28D) strain with overexpressed RimJ has a doubling time of 48 min compared with 54 min when the mutant cells contain the same vector (pBAD) with no insert (Fig. 1A). Overexpresion of RimJ has no effect on the growth of wild-type cells (data not shown), suggesting that the normal dosage of RimJ is not limiting for growth in an otherwise wild-type background. This significant alteration in growth properties can be observed in liquid culture as demonstrated from the reported doubling times and on solid media as demonstrated from dilution plating experiments (Fig. 1). These findings indicate that increased dosage of RimJ is sufficient to suppress the cold sensitivity of S5(G28D).

### Deletion of *rimJ* exacerbates the growth defect of S5(G28D)

If there is a functional link between RimJ overexpression and the S5(G28D) protein, one prediction would be that deletion of *rimJ* should alter the growth of the S5(G28D) strain. To study this genetic link, a strain bearing a deletion of *rimJ (JW1053)* and the S5(G28D) mutation was constructed and the phenotype of the double mutant was analysed. Examination of doubling times revealed a growth defect of the [S5(G28D)Δ*rimJ*] double mutant strain relative to the parental strains, S5(G28D) or Δ*rimJ*. At reduced temperature (20°C), growth is virtually undetectable for both [S5(G28D)Δ*rimJ*] and S5(G28D) strains (doubling time of > 300 min). At the permissive temperature (37°C), the double mutant strain had an exaggerated doubling time (69 min) as compared with S5(G28D) (44 min) and Δ*rimJ* (36 min). This significant alteration in growth properties can be also observed on solid media (Fig. 1C). As expected, deletion of *rimJ* (JW1053) results in a slight growth defect compared with the parental BW25113 strain at all temperatures (Fig. 1C). This suggests that although deletion of *rimJ* is tolerated in a S5(G28D) background, this double mutation is deleterious and that the suppression observed with RimJ overexpresson is not merely non-specific.

### Overexpression of RimJ alleviates the ribosome defects associated with S5(G28D)

**Fig. 2:**
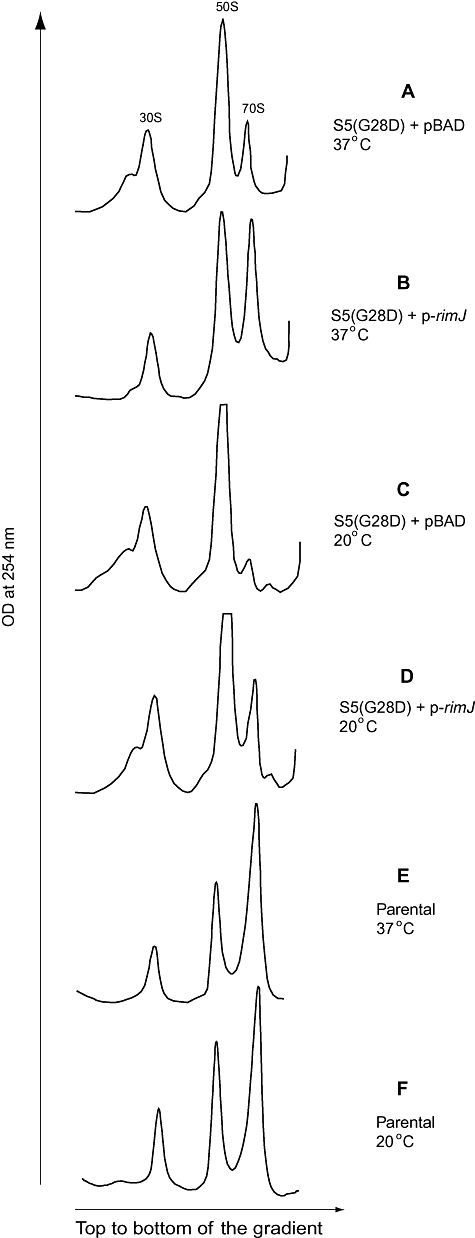
Overexpression of RimJ alters ribosome profiles of the S5(G28D) strain. Sucrose gradient sedimentation analysis of ribosomes isolated from the S5(G28D) mutant strains with empty pBAD (pBAD) and RimJ overexpressed (p-*rimJ*). Positions of the 30S and 50S subunits and 70S ribosomes are indicated. Ribosome analysis of S5(G28D) strain with pBAD grown at 37°C (A), p-*rimJ* grown at 37°C (B), pBAD grown at 20°C (C), p-*rimJ* grown at 20°C (D). Ribosome analyses of the parental CSH142 strain grown at 37°C (E) or 20°C (F) are shown for comparison.

One likely cause of the growth defects observed in the S5(G28D) strain is the abnormal ribosomal subunit/functional ribosome ratio observed in this strain. Ribosomes from strains carrying the S5(G28D) mutation are characterized by an increase in free 30S and 50S subunits compared with functional 70S particles and an accumulation of precursor 30S particles, especially at the non-permissive temperature (Kirthi *et al*., 2006; Fig. 2). To begin to address the underlying cause of suppression, ribosomes isolated from the S5(G28D) strain with and without overexpression of RimJ were examined. Ribosome profiles from S5(G28D) carrying an empty pBAD expression plasmid, and therefore no extragenic suppressor (Fig. 2, panels A and C) are similar to S5(G28D) alone (Kirthi *et al*., 2006). When RimJ expression is induced (Fig. 2, panels B and D), the profiles are altered and become more similar to those of the parental strain (Fig. 2, panels E and F) than to those observed in the mutant strain (Fig. 2, panels A and C). Concomitant with a slight change in the free subunits, increased amounts of 70S ribosomes are observed in profiles from the S5(G28D) + p*-rimJ* strain as compared with what is found in the S5(G28D) + pBAD strain (Fig. 2, panels A–D). RimJ induction appears to only slightly alter the free 30S subunit peaks (Fig. 2, compare panel C with D) and the extent of this change is temperature-dependent. It is possible that the pre-30S particles containing S5(G28D) which are formed prior to RimJ induction (see Experimental procedures) are ‘dead-end’ complexes and that RimJ cannot aid in their maturation. However, given the increase in functional 70S ribosome populations, and the concomitant decrease in doubling time, it seems likely that RimJ is able to aid in assembly of 30S subunits. Thus, while rescue in ribosome biogenesis of S5(G28D) strain is not complete at this level of RimJ induction, it appears that overexpression of RimJ contributes to the process. Additionally, deletion of *rimJ* results in slightly altered ribosome profiles relative to wild-type cells (B. Roy-Chaudhuri and G.M. Culver, unpubl. results), further indicating that RimJ plays a role in ribosome biogenesis.

### Overexpression of RimJ alleviates the translational fidelity defects associated with S5(G28D)

**Fig. 3:**
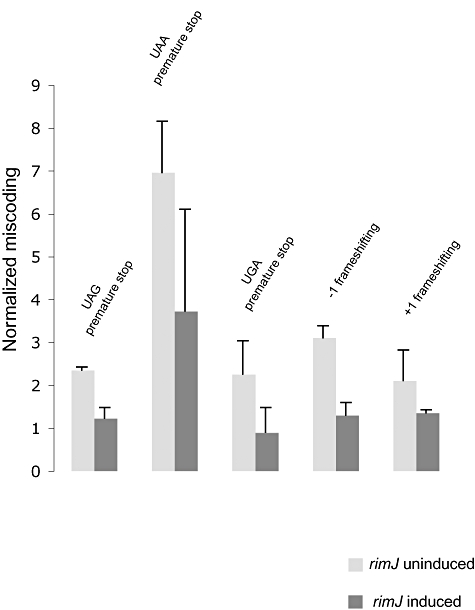
RimJ rescues translational miscoding of S5(G28D). Nonsense suppression and frameshifting as monitored by β-galactosidase activity in S5(G28D) in the presence (dark bars) and absence (light bars) of RimJ overexpression. Data are presented as normalized values and are an average of at least three independent experiments. Units of normalized β-galactosidase activity for the Δ*lacZ* S5(G28D) strain with various pSG constructs (Kirthi *et al*., 2006) and p-*rimJ* are indicated as ratios to those observed in the isogenic MC252 strain.

Given that the G28D mutation in S5 results in a significant increase in errors in interpreting the genetic code (Kirthi *et al*., 2006), we next tested if the suppression effect of RimJ could be extended to rescue this defect in translational fidelity. Plasmids carrying either the wild-type *lacZ* gene or the *lacZ* gene with mutations in the 5′ end enable miscoding to be assessed by monitoring β-galactosidase activity *in vivo* (O'Connor *et al*., 1997), using both parental and mutant strains (Fig. 3). Induction of RimJ expression in the S5(G28D) background results in reduced levels of miscoding as compared with what is observed in S5(G28D) cells in the absence of *rimJ* induction (Fig. 3). While the suppression of fidelity defects is not complete, the errors are reduced significantly and approach wild-type levels (Kirthi *et al*., 2006). Thus RimJ may act to restore growth rates near to wild-type in the presence of S5(G28D) by increasing not only the population of ribosomes (Fig. 2) but also elevating the translational accuracy of the ribosomes that are produced (Fig. 3).

### Mutation of glycine to aspartate in S5 does not alter the acetylation state of S5

In an attempt to dissect the manner by which extra copies of *rimJ* can suppress defects associated with S5(G28D), the known function of RimJ was evaluated. RimJ is the *N*-acetyltransferase of S5 (Cumberlidge and Isono, 1979; Yoshikawa *et al*., 1987) and has the well-conserved NAT (*N*-acetyltransferase) domain (Neuwald and Landsman, 1997; Vetting *et al*., 2005). Thus one possible scenario for suppression is that S5(G28D) is non-or under-acetylated and that overexpression of RimJ could increase acetylation of S5(G28D). This would offer a simple and plausible means by which RimJ can modulate the phenotypes associated with this S5 mutation. To determine the acetylation status of S5(G28D) in 30S subunits, mass spectrometry of the 30S subunit proteins isolated from the S5 mutant strain was performed. Both MALDI-TOF and ESI spectrometry were performed and revealed similar results (Table 1 and data not shown). Mass analysis was carried out for cells grown at both the permissive and non-permissive temperatures. Under both conditions, very similar results were obtained; however, for simplicity, only the mass analysis at the permissive temperature has been shown (Table 1). Mass analysis revealed that the majority (at least 90%) of S5 protein in both wild-type and S5(G28D) 30S subunits (Table 1) are acetylated even in the absence of RimJ overexpression. Use of a Δ*rimJ* strain (CWZ387) (White-Ziegler *et al*., 2002) confirms that RimJ is indeed responsible for S5 acetylation and that unacetylated S5 can readily be distinguished from its acetylated counterpart by mass spectrometry analysis (Table 1). These findings suggest that suppression is not directly dependent on the acetylation state of S5 and that RimJ may have an additional function *in vivo*.

**Table 1:** Calculated molecular mass (Arnold and Reilly, 1999) and that of the most prominent species of S5 determined by MALDI-TOF mass spectrometry.

Protein	Sequence mass (Da)	Expected mass (Da; acetylated)	Experimental mass (Da)	Acetylation status
Wild-type S5 in CSH142	17472.3	17514.8	17515.0	Acetylated
S5(G28D) in S5(G28D) strain	17530.2	17572.7	17574.0	Acetylated
Wild-type S5 in Δ*rimJ*	17472.3	17514.8	17473.9	Not acetylated
Wild-type S5 in Δ*rimJ* + pBAD	17472.3	17514.8	17473.8	Not acetylated
Wild-type S5 in Δ*rimJ* + p-*rimJ*	17472.3	17514.8	17509.2	Acetylated
Wild-type S5 in Δ*rimJ* + p-*rimJ*(C54A, C105A)	17472.3	17514.8	17471.1	Not acetylated
S7 in CSH142	19888.0	NA	19890.9	Not acetylated

R-protein S7 has been analysed for comparison.

NA, not applicable.

### Suppression by RimJ does not require its acetyltransferase activity

**Fig. 4:**
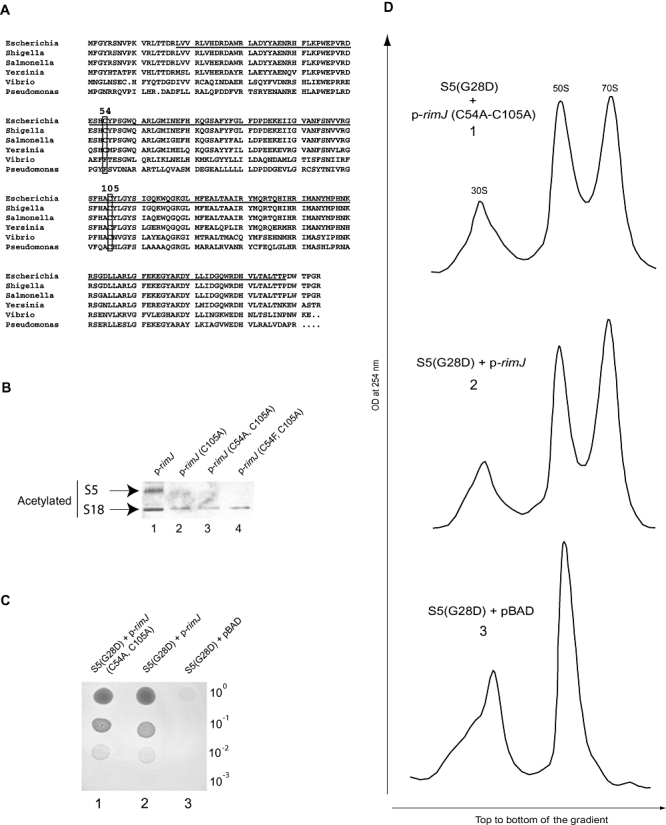
RimJ can suppress defects associated with S5(G28D) independent of its acetyltransferase activity. Overexpression of wild-type RimJ in the Δ*rimJ* or S5(G28D) background by induction of p*-rimJ.* Mutant RimJ proteins were overexpressed from p*-rimJ*(C105A), p*-rimJ*(C54A, C105A) and p*-rimJ*(C54F, C105A). Numbers and letters after the cysteines (C) correspond to positions in *E. coli* RimJ sequence that have been mutated to alanine (A) or phenylalanine (F). A. Multiple alignment of RimJ sequences and positions of C54 and C105. Sequence comparisons of RimJ from various prokaryotic species. The NAT domain in *E. coli* RimJ sequence is underlined. C54 and C105 residues are boxed. The RimJ sequences used in sequence alignment and their accession numbers in parenthesis are as follows. Escherichia: *Escherichia coli* (NP_415584), Shigella: *Shigella flexneri* (P0A950), Salmonella: *Salmonella enterica* (NP_455660), Yersinia: *Yersinia pestis* (CAL20676), Vibrio: *Vibrio cholerae* (YP_001216873), Pseudomonas: *Pseudomonas aeruginosa* (YP_791309). B. Western analysis for acetylated proteins in 30S subunit proteins (TP30) obtained from the Δ*rimJ* strain (CWZ387) with overexpression of various constructs: (1) p*-rimJ* (2) p*-rimJ*(C105A) (3) p*-rimJ*(C54A, C105A) (4) p*-rimJ*(C54F, C105A). C. 10-fold serial dilutions of S5(G28D) with various constructs at the non-permissive temperature, 20°C: (1) p*-rimJ*(C54A, C105A) (2) p*-rimJ* (3) empty pBAD. D. Sucrose gradient sedimentation analysis of ribosomes from S5(G28D) grown at the non-permissive temperature, 20°C, with the constructs: (1) p*-rimJ*(C54A, C105A) (2) p*-rimJ* (3) empty pBAD. Positions of the 30S and 50S subunits and 70S ribosomes are indicated.

To explore the requirement of the acetyltransferase function of RimJ for suppression of phenotypes associated with the S5(G28D) mutation, RimJ mutants that lack acetyltransferase activity were constructed. Cysteine residues have been implicated in the enzymatic activity of many acetyltransferases (Dupret and Grant, 1992; Watanabe *et al*., 1992; Pompeo *et al*., 1998; Dyda *et al*., 2000). Multiple alignment of the acetyltransferase domain sequences of RimJ from various species reveals that two conserved cysteines might be important for its enzymatic activity (Fig. 4A). Site-directed mutagenesis was carried out on p*-rimJ* to change the two cysteines at positions 54 and 105 in *E. coli* RimJ to alanine or phenylalanine residues to create four different singly and doubly substituted proteins [RimJ(C54F), RimJ(C105A), RimJ(C54F, C105A), or RimJ(C54A, C105A)]. To determine if these mutations were sufficient to obliterate acetyltransferase function, the mutant RimJ proteins were overexpressed in the *rimJ* deletion strain (CWZ387; White-Ziegler *et al*., 2002), and antibodies that recognize acetylated proteins were used to assess S5 acetylation. When wild-type RimJ is overexpressed from p*-rimJ* in CWZ387, a band corresponding to acetylated S5 is observed. In addition to S5, there is another small subunit r-protein, S18, which is acetylated by a separate enzyme, rimI (Isono and Isono, 1980; Yoshikawa *et al*., 1987) and this acetylated protein is also recognized by the same antibody (Fig. 4B, lane 1). When any of the mutant RimJ proteins is overexpressed in CWZ387, the band corresponding to acetylated S5 is not observed (Fig. 4B, lanes 2–4) while the band corresponding to acetylated S18 is observed (Fig. 4B, lanes 2–4). Furthermore, to confirm the acetyltransferase status of the mutant forms of *rimJ*, mass analysis of 30S r-proteins isolated from the Δ*rimJ* grown at 37°C and carrying either the empty vector, wild-type RimJ or RimJ(C54A, C105A) constructs revealed that the majority of S5 was unacetylated when either the empty vector or RimJ(C54A, C105A) was induced. However, S5 was clearly acetylated when wild-type RimJ was induced (Table 1). These results indicate that the cysteine-substituted RimJ proteins lack S5 acetyltransferase activity and thus provide a tool to assess the role of acetylation in *rimJ* suppression.

One acetyltransferase-deficient clone of *rimJ*, RimJ(C54A, C105A), was used for cold sensitivity suppression analysis. Overexpression of RimJ(C54A, C105A) in S5(G28D) at the non-permissive temperature results in similar growth as seen with wild-type RimJ (Fig. 4C), suggesting that the acetyltransferase-deficient RimJ can suppress the cold sensitivity of S5(G28D). Furthermore, analysis of the ribosome profiles (Fig. 4D) indicates that as observed when wild-type RimJ is overexpressed (Fig. 2D), an increased amount of 70S particles is formed with the slight changes in free 30S and 50S subunits upon overexpression of RimJ(C54A, C105A) when compared with empty pBAD (Fig. 4D). Hence, it appears that the acetyltransferase-deficient RimJ mutant acts in a similar fashion as wild-type RimJ to suppress the cold sensitivity and other phenotypes associated with S5(G28D). These findings indicate that RimJ can suppress the S5(G28D) defects independent of its acetyltransferase function and that RimJ functions in a previously unknown capacity related to ribosome assembly and function *in vivo*.

### RimJ associates with assembling 30S subunits

**Fig. 5:**
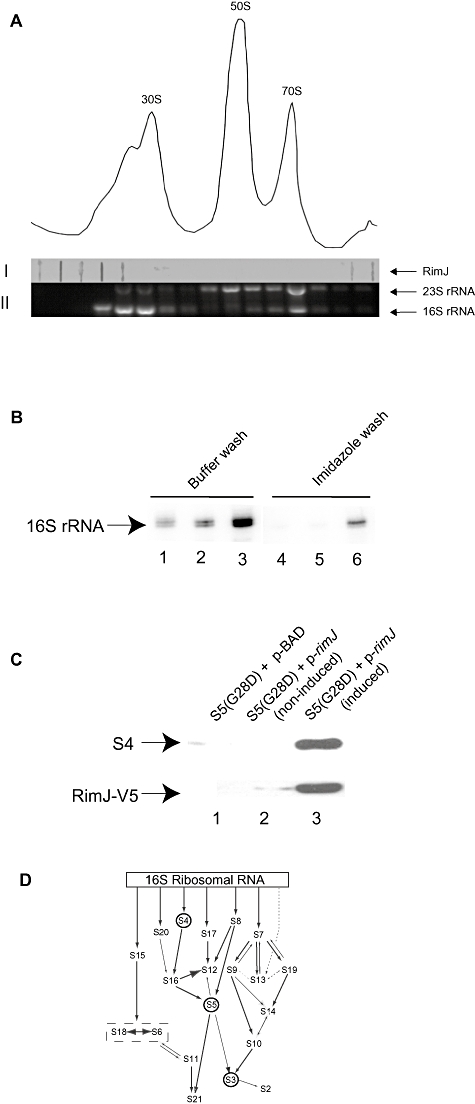
RimJ associates with assembling 30S subunits *in vivo*. A. Analysis of ribosomal profile of S5(G28D) grown at the non-permissive temperature with induction of p-*rimJ*. Fractions from across this gradient were analysed by (I) slot-blot for RimJ or (II) gel electrophoresis for 23S rRNA and 16S rRNA to determine location of ribosomal particles. B. Primer extension analysis of 16S rRNAs extracted from immunoprecipitated RNPs with pBAD (Lanes 1, 4), non-induced p-*rimJ* (Lanes 2, 5) and induced p-*rimJ* (Lanes 3, 6) in S5(G28D) cells. Ni-NTA beads were washed with B-150 buffer (Lanes 1–3) or imidazole (Lanes 4–6). C. Western analysis of RNPs immunoprecipitated with pBAD (Lane 1), non-induced p-*rimJ* (Lane 2) and induced p-*rimJ* (Lane 3) in S5(G28D) cells for S4 and RimJ-V5 (indicated by arrows). D. Modified *in vitro* assembly map for 30S subunits (Grondek and Culver, 2004). Arrows indicate dependency between components for association with 16S rRNA. Positions of S4, S5 and S3 are indicated by circles.

To test the association of RimJ with ribosomal particles as has been shown for many assembly factors, fractions were collected across the ribosome profiles of S5(G28D) with RimJ overexpressed at the non-permissive temperature. Immunochemical analysis revealed RimJ in fractions containing free pre-30S subunits, but apparently not in mature 30S subunits or in 50S subunits or 70S particle containing fractions (Fig. 5A). These results indicate that RimJ exerts its role on the RNP and not solely on free S5. Although we see RimJ in some fractions that are denser the 70S ribosome peak, this fraction of RimJ does not co-sediment with any rRNA (i.e. polysomal particles) and the amount diminishes with shorter induction of p-*rimJ* (data not shown). Subunit dissociation and polysome analysis of ribosomes from S5(G28D) strain with RimJ overexpression further suggest that there is no stable interaction of RimJ with polysomes (data not shown). Thus it appears that these fast sedimenting molecules are aggregates of RimJ that can be observed upon extensive overexpression.

The stability of interaction of RimJ with pre-30S particles was further studied by isolating pre-30S particles from S5(G28D) strain with RimJ overexpressed and re-sedimenting these particles through gradients containing increasing concentrations of ammonium chloride. RimJ was found to be associated with pre-30S subunits at different concentrations of NH_4_Cl tested, even at 0.5 M NH_4_Cl (data not shown), suggesting that the co-sedimentation of RimJ with pre-30S subunits is not fortuitous but likely due to a valid interaction. To augment our hypothesis that RimJ binds to 16S rRNA containing RNPs *in vivo*, we performed immunoprecipitations using RimJ. Analysis of the precipitated complexes for RNA revealed that RimJ associates with 16S rRNA (Fig. 5B) but not with 23S rRNA (data not shown). Analysis of the immunoprecipitated complexes for specific r-proteins indicates the presence of S4 (Fig. 5C) but not S3 (data not shown). R-protein S4 is an early binding protein whereas S3 is a late assembling 30S subunit r-protein (Fig. 5D), indicating that RimJ binds to the assembling 30S RNP at an intermediate stage of assembly. This is consistent with the observation that RimJ associates with pre-30S subunits on the ribosome profile (Fig. 5A). These findings reveal that RimJ can associate with pre-subunit RNPs and it is likely via this interaction that it exerts its function in the biogenesis cascade.

## Discussion

In our previous work, we identified a unique point mutation within S5 which results in a change of a glycine residue to an aspartate at a highly conserved position [S5(G28D); Kirthi *et al*., 2006]. The S5(G28D) mutation leads to defects in ribosome biogenesis and translational fidelity (Kirthi *et al*., 2006). However, this substitution is distinct from the previously characterized S5 ram mutations (Piepersberg *et al*., 1975a,Piepersberg *et al*., 1975b), as the S5(G28D) mutant, unlike the other ram mutants, is cold sensitive, a hallmark of defects in ribosome biogenesis. Additionally, this S5 mutation is remote from the structural clustering of other known ram mutations including S4 ram mutations (Donner and Kurland, 1972; Funatsu *et al*., 1972; van Acken, 1975; Ogle *et al*., 2002; Ogle and Ramakrishnan, 2005), and thus appears to be a new ram mutant. To address the functional significance of this S5 mutant, extragenic suppressors were selected by their ability to alleviate the cold sensitivity of S5(G28D) mutant strain. RimJ, which is the N-terminal acetyltransferase (NAT) of S5 (Yoshikawa *et al*., 1987; Polevoda and Sherman, 2003), was identified as a suppressor and our work demonstrates that overexpression of the wild-type protein can alleviate phenotypes associated with S5(G28D). Additionally, we have shown that the ability of RimJ to suppress S5(G28D) defects is independent of its acetyltransferase function and therefore have identified an additional role of this protein *in vivo*.

Both r-proteins and rRNAs can be modified, however, the functional significance of these modifications remains unclear. Acetylation as a modification is found at the N-termini of r-proteins S5, S18 and L12 (Cumberlidge and Isono, 1979; Isono and Isono, 1980; Isono and Isono, 1981), and these modifications are carried out by three separate NATs, RimJ, RimI and RimL respectively (Yoshikawa *et al*., 1987; Tanaka *et al*., 1989). The NAT enzyme family, with the putative acetyl-CoA-binding motifs A–D, is ubiquitous in all kingdoms, suggesting an underlying importance to cell viability (Neuwald and Landsman, 1997; Dyda *et al*., 2000; Polevoda and Sherman, 2002). However, like many rRNA modification genes (Lu and Inouye, 1998; Tan *et al*., 2002; Gutgsell *et al*., 2005; Baba *et al*., 2006), *rimJ* is not essential for viability (White-Ziegler *et al*., 2002; Baba *et al*., 2006). Thus, S5 in its unacetylated state must be sufficient for ribosome assembly and function. Again, as for rRNA modifications, it is possible that modification of this r-protein aids in fine-tuning the structural and functional capacity of the translational machinery. Also, the viability of the *rimJ* deletion strain indicates that the additional function of RimJ uncovered in this work is not essential. Other factors associated with ribosome biogenesis in *E. coli* have also been shown to be dispensable for growth (Dammel and Noller, 1995; Bylund *et al*., 2001; Charollais *et al*., 2003; 2004; El Hage and Alix, 2004), suggesting that *E. coli* ribosome biogenesis is robust and that there are possibly redundant pathways. This would allow the cell to compensate for loss of specific assembly factors and thus increase the overall likelihood of survival.

Many factors implicated in ribosome biogenesis are also involved in multiple cellular functions. The *E. coli* DEAD-box proteins (Silverman *et al*., 2003; Iost and Dreyfus, 2006) which have been shown to participate in ribosome biogenesis (CsdA, SrmB) also participate in mRNA processing and decay as well as in translation initiation (Iost and Dreyfus, 1994; Lu *et al*., 1999; Prud'homme-Genereux *et al*., 2004). Similarly, Era, which is one of the most-studied bacterial GTPases, has roles in cell cycle and cell division (Gollop and March, 1991), RNA binding (Johnstone *et al*., 1999) and is involved in carbon and nitrogen metabolism (Lerner and Inouye, 1991; Powell *et al*., 1995). Likewise, RimJ can act as a negative repressor of pyelonephritis-associated pili (Pap) expression in uropathogenic *E. coli* (White-Ziegler and Low, 1992; White-Ziegler *et al*., 2002). Thus, it may play an important role in adapting uropathogenic bacteria to changing environments. However, it is unknown if acetylation of S5 might be involved in downregulating *papBA* transcription (White-Ziegler *et al*., 2002) or similar to suppression of S5(G28D)-associated phenotypes if this role is also independent of acetyltransferase activity. The mechanism by which RimJ acts in cellular processes outside of acetylation will be interesting to dissect. Other than a few N- and C-terminal amino acids in RimJ (194 residues), the remaining 171 amino acids form the NAT domain (Fig. 4A, ExPASy-PROSITE search). There is no obvious or readily discernible functional domain, other than the acetyltransferase, in RimJ and thus it is not clear how RimJ facilitates these additional processes. Given the level of homology with other NATs (see above) it is likely that acetylation of S5 was the initial function of RimJ. Once RimJ had been recruited to the biogenesis cascade, the second biogenesis role identified herein may have evolved. This would allow *E. coli* to make optimal use of its somewhat limited genome and thereby allow optimization of ribosome biogenesis.

How is RimJ related to ribosome biogenesis? It has been shown that unacetylated S5 binds to the assembling RNP at a faster rate than acetylated S5 (Talkington *et al*., 2005), suggesting that acetylation of S5 could occur once S5 has associated with the 16S rRNA-containing RNP. This association could then augment subsequent changes or preclude some non-productive interactions and thus aid in the assembly process. RimJ might bind to the assembling 30S subunits through or adjacent to S5. We show that RimJ associates with assembling 30S subunits devoid of S3 (Fig. 5 and data not shown) *in vivo*. This is of particular interest as S4 is a primary binding protein (Grondek and Culver, 2004) to 16S rRNA in the assembly pathway of 30S subunits (Fig. 5D). S3 binds much later in the hierarchical order of binding of small subunit proteins. Additionally, our previous work demonstrated that S5(G28D) did not greatly alter S3 association (Kirthi *et al*., 2006). This might indicate the binding of RimJ at an intermediate step of 30S subunit maturation and suggest that acetylation of S5 and the additional function of RimJ could be occurring at the same stage of ribosome biogenesis.

## Experimental procedures

### Strains

Strains S5(G28D), Δ*lacZ* S5(G28D), JW1053 and CWZ387 have been described previously (White-Ziegler *et al*., 2002; Baba *et al*., 2006; Kirthi *et al*., 2006). The double mutant [S5(G28D)Δ*rimJ*] was created by transducing JW1053 to kanamycin and spectinomycin resistance using a phage P1 lysate of S5(G28D). The deletion of *rimJ* in this strain was confirmed by PCR amplification and the presence of the S5(G28D) mutation was ascertained by S5 gene (*rpsE*) amplification and subsequent sequencing using *rpsE*-specific primers.

### Selection of extragenic suppressors of the cold-sensitive S5(G28D) strain

A high-copy *E. coli* pUC-based genomic library (a kind gift of G. Phillips, Iowa State University) was transformed into the S5(G28D) strain and colonies that could grow at the non-permissive temperature (20°C) on 2XYT plates containing ampicillin (100 μg ml^−1^) and spectinomycin (60 μg ml^−1^) were identified.

### Doubling times and dilution plating

Cells were grown at 37°C (permissive temperature) to lag phase or grown at 37°C for 60 min prior to being shifted to 20°C (non-permissive temperature). Cell growth was continued for another 30 min at the non-permissive temperature before adding 0.2% arabinose, when appropriate. Optical density at 600 nm was measured every 30 min during the course of the growth (at least a total of 7 h) to obtain growth curves and determine the doubling times.

For dilution plating, an equal number of cells from overnight cultures grown in LB plus appropriate antibiotics at 37°C were subcultured into fresh LB and grown at 37°C to an OD of ∼0.3. Ten-fold serial dilutions were made using fresh LB and plated on LB plates plus appropriate antibiotics and 0.2% arabinose, when required. Plates were incubated at 37°C overnight or at the non-permissive temperature for at least 48 h.

### Ribosome analysis

For analysis of ribosome profiles, cell growth was carried out as done for estimation of doubling times (mentioned above). Ribosome analysis was essentially carried out as described (Maki *et al*., 2002). The ribosomes were analysed by sucrose gradient sedimentation using 10–40% sucrose gradients in polysome buffer [20 mM Tris-HCl (pH 7.8), 10 mM magnesium chloride and 100 mM ammonium chloride] in a SW41 rotor (25 000 r.p.m.) for 17 h at 4°C. The speed and time were optimized to enable a better separation of the 30S and the pre-30S peak, therefore not allowing polysomes to be readily observed.

To localize RimJ in the ribosome profiles, fractions of 1 ml were collected across the profiles of S5(G28D) with p-*rimJ* induced at the non-permissive temperature. Slot-blot analysis using a V5 antibody (against recombinant RimJ) was performed with amounts of RNA (OD measured at 260 nm) equalized. 16S rRNA and 23S rRNA were used as markers for 30S and 50S subunits, respectively, and analysed by agarose gel electrophoresis.

### Cloning and expression of *rimJ*

The *rimJ* gene was PCR amplified from *E. coli* (CSH142) genomic DNA using the primers: 5′-CAGGCTATTCATATGTTTGGCTATCGCAGTAACGTG-3′ and 5′-TCCGCGGCTGAATTCTTAGCGGCCGGGCGTCCA-3′.

It was ultimately inserted into the pBAD-DEST49 vector by GATEWAY technology (Invitrogen). Expression of *rimJ* was induced by a final concentration of 0.2% arabinose. The construct carried a V5-epitope for indirect detection of the recombinant RimJ and a polyhistidine tag that was utilized for immunoprecipitation.

### DNA preparation and sequencing

DNA sequencing of the suppressor element of S5(G28D) was performed using pUC/M13 sequencing primer (5′-TGTAAAACGACGGCCAGT) and reverse (M13) primer (5′-CAGGAAACAGCTATGACC-3′) at the Iowa State University sequencing facility. Site-directed mutagenesis to create substitutions of cysteine residues in p-*rimJ* was conducted using QuickChange kit (Stratagene). The primers used for site-directed mutagenesis were:

- (C54F) sense primer: 5′-GACGAAAGCCACTTTTATCCATCAGGC-3′ and the corresponding antisense primer;
- (C54A) sense primer: 5′-GACGAAAGCCACGCCTATCCATCAGGC-3′ and the corresponding antisense primer; and
- (C105A) sense primer: 5′-CTTTTCATGCCGCCTATCTCGG-3′ and the corresponding antisense primer.

The underlined codons correspond to the substituted amino acids.

### Mass spectrometry

30S subunits were collected by sucrose gradient sedimentation of cell lysates from wild-type CSH142 or S5(G28D) cells grown at both 37°C and 20°C. For the CWZ387 strain in the presence of pBAD, p-*rimJ* or p-*rimJ*(C54A, C105A), growth was carried out at 37°C. MALDI and ESI samples were prepared by adding two volumes of 1 M magnesium acetate and glacial acetic acid mixture (1:20, v/v) to the 30S subunits and the extracted r-proteins (Nierhaus, 1990) were desalted. Mass analysis was performed at the Iowa State University Proteomics facility.

### β-Galactosidase assay

The Δ*lacZ* S5(G28D) strain used here was available from our previous work (Kirthi *et al*., 2006). This strain was cotransformed with all the *lacZ* plasmids for fidelity (pSG constructs as discussed previously: O'Connor *et al*., 1997) and p*-rimJ*. The various pSG constructs carry in-frame stop codons or frameshifts in the *lacZ* sequence.

Cells to be assayed for the activity of β-galactosidase, as described previously (O'Connor *et al*., 1997), were grown in LB medium containing 12.5 μg ml^−1^ of tetracycline and 100 μg ml^−1^ ampicillin and in the presence or absence of 0.2% arabinose. β-Galactosidase activity from the plasmid encoding wild-type *lacZ* in the wild-type strain, MC252 (O'Connor *et al*., 1997) and the isogenic mutant strain, Δ*lacZ* S5(G28D) strain (Kirthi *et al*., 2006), with *rimJ* induced or uninduced, were used for normalization. Units of normalized β-galactosidase activity for the Δ*lacZ* S5(G28D) strain with various constructs and p-*rimJ* were reported as ratios to those observed in the isogenic MC252 strain.

### Western analysis to determine the acetylation status of S5

Total Protein 30 (TP30) was isolated from the 30S subunits (Nierhaus, 1990) of CWZ387 strain (a kind gift of Christine A. White-Ziegler, Smith College) in the presence of overexpressed wild-type RimJ or mutant forms of RimJ. Similar amounts of total proteins (TP30) were loaded and were resolved using 15% Next Gel (Amresco) followed by immunoblotting with a rabbit antibody against acetylated proteins (Abcam; ab193).

### Immunoprecipitation of 30S complexes

Cell extracts from S5(G28D) cells with pBAD, p-*rimJ (* non-induced) or overexpressed RimJ (p-*rimJ* induced) were prepared in B-150 buffer [150 mM NaCl, 50 mM Tris-HCl (pH 7.4), 0.2% Triton-X, 1X Roche-Complete Protease Inhibitor). Cleared extracts containing ∼3 mg ml^−1^ total protein were incubated with Ni-NTA Agarose beads (Qiagen) for 3 h at 4°C. After washing six or more times with B-150 or twice with 50 mM imidazole, bound complexes were analysed for 16S rRNA or 23S rRNA by primer extension or by Western blotting using either S3, S4 or V5 (against recombinant RimJ) antibodies.
